# Investigation Report of cVDPV2 Outbreak in Bokh Woreda of Dollo Zone, Somali Regional State, Ethiopia

**DOI:** 10.1155/2020/6917313

**Published:** 2020-08-26

**Authors:** Diriba Sufa, Urge Gerema

**Affiliations:** ^1^Ethiopian Public Health Institute (EPHI), Center for Public Health Emergency Management (cPHEM), Addis Ababa, Ethiopia; ^2^Department of Biomedical Sciences, Division of Anatomy, College of Medical Sciences, Institute of Health Sciences, Jimma University, Jimma, Ethiopia

## Abstract

**Background:**

Poliovirus isolates detected in persons or in the environment can fall into three major categories: wild, Sabin and Sabin-like, or vaccine-derived. Detection of wild or vaccine-derived poliovirus may constitute an emergency, which can be categorized as an event that can lead to an outbreak, depending on characteristics of the isolate and the context in which it appears. The aim of the study was investigation report of cVDPV2 outbreak in Bokh woreda of Dollo Zone, Somali regional state, Ethiopia.

**Methods:**

A team of experts drawn from different organizations was deployed to Bokh woreda to make detailed field investigation from May 25 to June 17, 2019. By using standard World Health Organization polio outbreak investigation checklist, document review of surveillance, immunization, and clinical data related to the case was made. Key informant's interview was made to health professionals, managers, parents of case, woreda and kebele leaders, religious leaders, and HEWs related to acute flaccid paralysis outbreak.

**Result:**

The notified AFP case was a 39-month-old female from Angalo kebele of Bokh woreda, Dollo Zone. On 19th May 2019, the patient developed high grade fever and was taken to Angalo Health Post on 20th May 2019. As per the examination by a health extension worker, the child had high grade fever and neck stiffness with preliminary diagnosis of meningitis for which ceftriaxone injection was prescribed. Contact sample was taken from three children on 28th May 2019 and 29th May 2019 and was sent to Addis Ababa National Polio Laboratory. All contact stool samples were found to be positive for poliovirus type 2 and referred for sequencing in National Institute of Communicable Diseases (NICD), South Africa, the Regional Polio Reference Laboratory. *Conclusion and Recommendation*. The clinical presentation of the cases is compatible with poliovirus infection, improving the quality and coverage of supplementary polio immunization activities through proper planning; strict supervision and follow-up can reduce the occurrence of acute flaccid paralysis.

## 1. Introduction

Poliovirus isolates detected in persons or in the environment can fall into three major categories: wild, Sabin and Sabin-like, or vaccine-derived [1–3]. Detection of wild or vaccine-derived poliovirus may constitute an emergency, which can be categorized as an event that can lead to an outbreak depending on characteristics of the isolate and the context in which it appears [2, 4–6]. Genetically, vaccine-derived poliovirus (VDPV) strains can emerge during vaccine use and spread in underimmunized populations, becoming circulating (cVDPV) strains and resulting in outbreaks of paralytic poliomyelitis [2, 7–10]. The global polio eradication was set up in 1988 when the World Health Assembly (WHA) passed a resolution to eradicate polio by the year 2000 [11, 12]. Due to many challenges, the goal set for 2000 was not met [12]. The global polio eradication initiatives developed the polio eradication and endgame strategic plan covering the period 2013 to 2018, aiming at a polio-free world by 2018 [13, 14]. The basic strategies for eradicating polio include the following: (i) immunization of every child aged less than one year with at least three doses of oral polio vaccine (OPV); (ii) national immunization days (NIDS) in which all children less than five years of age receive extra doses of OPV on two days separated by at least 28 days; (iii) surveillance for acute flaccid paralysis to identify all reservoirs of wild poliovirus transmission; and (iv) extensive house to house immunization mopping-up campaigns in the final stages in areas where wild poliovirus transmission persists [12, 15, 16]. In the prepolio eradication era, polio was generally endemic in many African countries with 1597 polio cases recorded in the continent in 1995 [17].

The implementation of the polio eradication strategies in Ethiopia started in 1996 [18]. Ethiopia was polio free for four years from January 2001 until December 2004. From December 2004 to February 2006, a total of 24 cases were confirmed from Tigray, Amhara, and Oromia regional states. In 2004, the surveillance performance for acute flaccid paralysis (AFP) at national level was above the targets as of February 2005 [17, 19]. We present the AFP cases from Angalo kebele of Bokh woreda, Dollo Zone, which is above the targets.

### 1.1. Immunization Service Status in the Woreda

Bokh woreda provides routine immunization service both by static and outreach strategies [20]. The trend of the RI coverage for the woreda showed rapid decline over the last four years. Data obtained from the Regional Health Bureau showed that the average SIA coverage of the woreda was 98%, 97%, 97.5%, and 97% in the year 2015, 2016, 2017, and 2019, respectively. Only five (16.7%) of under-five children reported to have received at least 3 doses of OPV during all rounds of polio SIAs [20]. The aim of the study was to present investigation report of cVDPV2 outbreak in Bokh woreda of Dollo Zone, Somali regional state, Ethiopia.

### 1.2. Case Presentation

The notified AFP case is a 39-month-old female from Angalo kebele of Bokh woreda, Dollo Zone. On 19th May 2019, the child developed high grade fever and was taken to Angalo Health Post on 20th May 2019. As per the examination by health extension worker, the child had high grade fever and neck stiffness with preliminary diagnosis of meningitis for which ceftriaxone injection was prescribed. Despite the administration of antibiotic injection for two days, the child become critically sick and started to have paralysis of the lower limb followed by weakness of the upper extremities. The case was then taken to Buhodle, Somaliland, for further diagnosis and management and notified as an AFP case from Somalia on 25th May 2019. Stool sample was obtained and taken to Nairobi, Kenya, on 27th May 2019. On 9th June 2019, it was reported, through World Health Organization (WHO), the laboratory results of the AFP case turned out to be positive for poliovirus-2. The WHO Somaliland polio team at Buhodle was notified about the case on 25th May 2019 and investigation started on 26th May 2019. The first and second stool samples were collected on 27th May 2019 and 28th May 2019, respectively, and sent to polio laboratory at Kenya Medical Research Institute (KEMRI) in Nairobi for laboratory investigation.

Contact sample was taken from three children on 28th May 2019 and 29th May 2019 and was sent to Addis Ababa National Polio Laboratory. All contact stool samples were found to be positive for poliovirus type 2 and referred for sequencing in National Institute of Communicable Diseases (NICD), South Africa, the Regional Polio Reference Lab. The ages of these close contacts are 56 months, 54 months, and 33 months. Of three contacts, only one child received one dose of OPV through RI. All the three contacts got one dose of OPV through SIA according to information from mothers of the children.

## 2. Discussion

The case here described represents cVDPV2 outbreak. cVDPV2 was clinically suspected because of the appearance of child, high grade fever, neck stiffness, and paralysis of the lower limb followed by weakness of the upper extremities. It was confirmed with isolation of cVDPV in stool specimens collected from the suspected case or from a close contact.

Global experiences showed that VDPV has shown increased trends in areas with low immunization coverage of both routine and SIA [11]. The incidence of type-2 strain of the VDPV is disproportionally higher than the other types [20]. Ethiopia switched providing tOPV as routine since March 2016 following eradication of type poliovirus from the world. Since then, a number of SIAs has been conducted in Ethiopia [21]. Absence of routine OPV vaccination and repeated uses of bOPV might have contributed to occurrence of VDPV in this case. Though family and community have positive attitudes towards and high demands for immunization, the services are not reaching them. Apart from population mobility, unreliable security, transport, and manpower shortages, lack of proper planning contributed to low immunization coverage.

Case detection and reporting trends are optimal. Though knowledge gaps exist among health workers at all levels on other causes of AFP, there are no unreported cases found. No missed cases were also found during house-to-house case searches and from in-depth inquiry of family and communities. It is worth-mentioning that community and its leaders are well aware of the signs and symptoms of the disease and are highly sensitive for reporting to the health system. The case was immediately taken to nearby health center. Two of the contact cases from household and neighborhood were not vaccinated by routine immunization. All contacts received one OPV dose from 2017 SIAs. The lab results of all contacts revealed to be positive for poliovirus-type two at the national polio laboratory and were sent for further investigation to South Africa. The newly found AFP cases have also not received any dose from RI and received SIA doses. It was also noted that there was no significant travel history to and from other woredas of the region and neighboring Somalia. The results of lab tests for both cases are pending at EPHI.

## 3. Conclusion

Based on the detailed case assessment, epidemiological, sociodemographic, health status including EPI, and surveillance investigation and laboratory findings, the investigation team comes up with the following conclusion.

As per the information collected from the parents and the locals, the family of the index case has been living in the area for more than seven years and has lived within the same kebele prior, which ensures the child is from Ethiopian side. The clinical presentation of the case is compatible with poliovirus infection.

## Data Availability

The datasets used and/or analyzed during the current study are available from the corresponding author on reasonable request.

## Abbreviations

AFP: Acute flaccid paralysis
bOPV: Bivalent oral polio vaccine
CBS: Community-based surveillance
cPHEM: Circulating Public Health Emergency Management
CVDPV2: Circulating vaccine-derived poliovirus
EPHI: Ethiopian Public Health Institute
EPI: Expanded program immunization
FGD: Focused group discussion
FMOH: Federal Ministry of Health
IDSR: Integrated Disease Surveillance and Response
KEMRI: Kenya Medical Research Institute
NIDS: National immunization days
OPV: Oral polio vaccine
PHEM: Public Health Emergency Management
PV2: Polio vaccine 2
RHB: Regional Health Bureau
RI: Routine immunization
SIA: Supplementary polio immunizations activities
tOPV: Trivalent oral polio vaccine
UNICEF: United Nations Children's Fund
VDPV: Vaccine-derived poliovirus
WHO: World Health Organization.
